# Supplementary material for: YB-1 Is a Novel Target for the Inhibition of α-Adrenergic-Induced Hypertrophy
Source: Int J Mol Sci. 2023 Dec 28;25(1):401. doi: 10.3390/ijms25010401 (PMC10778708; doi:10.3390/ijms25010401)
Supplement: Supplementary file 1 [file ijms-25-00401-s001.zip › ijms-2674099-supplementary.pdf]

Supplementary Material

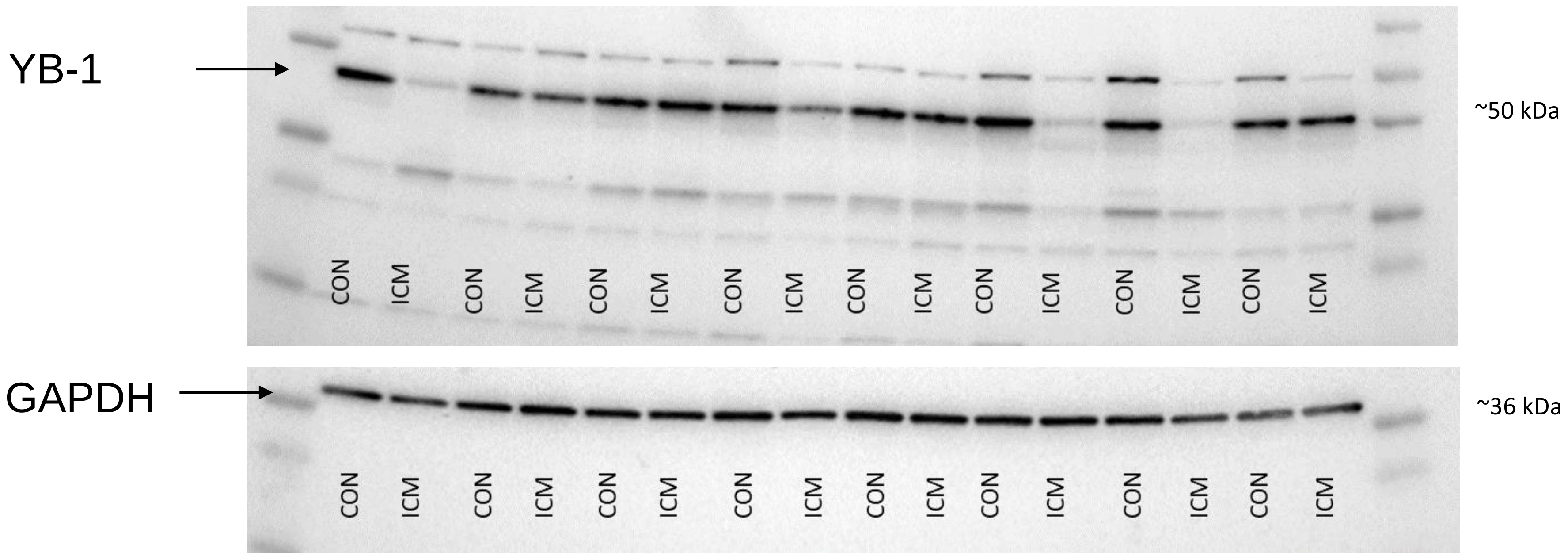

Figure S1: Complete blot with all human heart biopsy samples.

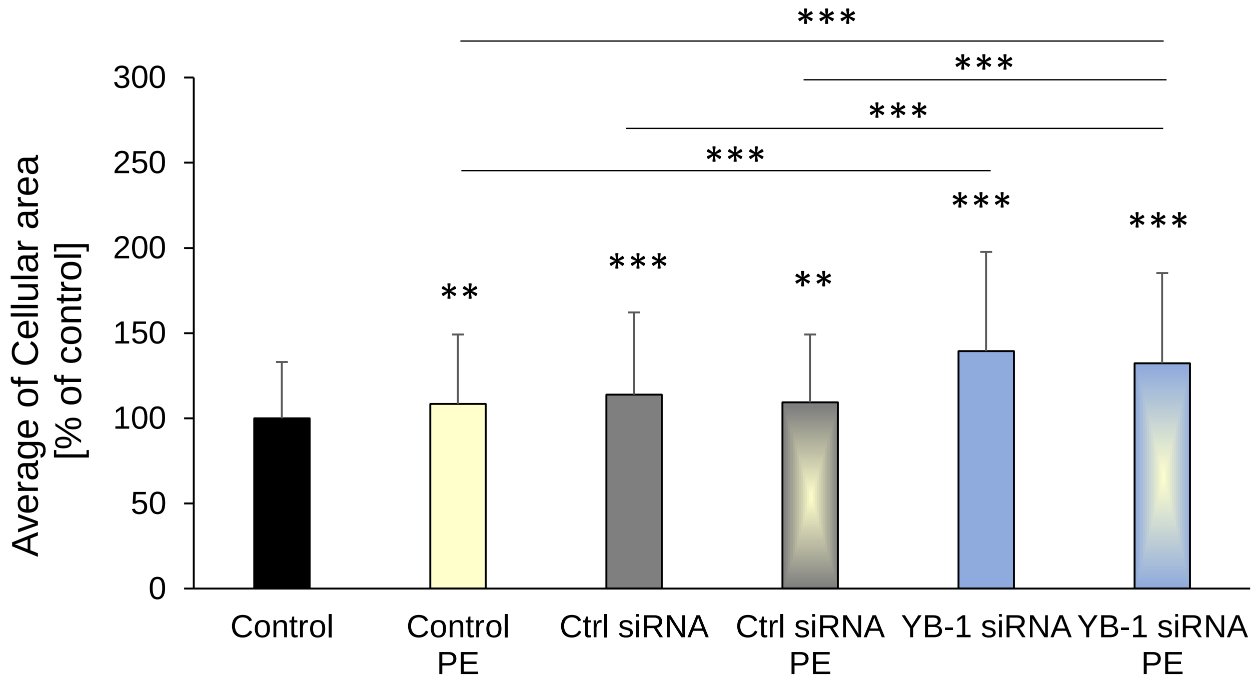

Figure S2: ***YB-1* downregulation in H9C2 cells leads to an increase in cell surface area.** Cardiomyocytes were transfected with *YB-1* siRNA or Ctrl siRNA or were non-transfected. Cells were photographed 48 hours after transfection with siRNA, preceded by 24 hours of PE (10 µM) stimulation (n =227 – 311 cells, 18 dishes of three individual cell passages). Data are presented as the means ± standard deviation. Key: **, $p < 0.01$; ***, $p < 0.001$.

Transcript ID	Gene symbol	Gene name	Off-target score
ENSRNOT00000007994.6	Gdap1	ganglioside-induced differentiation-associated-protein 1	-7.569
ENSRNOT00000086575.1	Slc25a23	solute carrier family 25 member 23	-7.512
ENSRNOT00000018988.4	Paqr5	progestin and adipoQ receptor family member 5	-7.408
ENSRNOT00000023354.7	Mycbp	Myc binding protein	-7.202
ENSRNOT00000089319.2	Lrrc75b	leucine rich repeat containing 75B	-7.19
ENSRNOT00000080859.1	Acot1	acyl-CoA thioesterase 1	-7.159
ENSRNOT00000072580.3	Ecscr	endothelial cell surface expressed chemotaxis and apoptosis regulator	-7.13
ENSRNOT00000079478.1	AABR07006311.1	LOC100910386 protein FAM111A-like	-7.024
ENSRNOT00000087595.1	AABR07068316.1	collagen type VI alpha 3 chain	-6.972
ENSRNOT00000026719.5	AABR07068316.1	collagen type VI alpha 3 chain	-6.972

Table S1: **Predicted siRNA off-targets.**
Top ten potential off-target genes with the greatest absolute value of the off-target score.

GO biological process	Fold Enrichment	FDR
pericardium development (GO:0060039)	2.92	4.41E-02
artery morphogenesis (GO:0048844)	2.15	9.53E-03
morphogenesis of an epithelial sheet (GO:0002011)	2.12	3.65E-02
intracellular receptor signaling pathway (GO:0030522)	1.99	2.15E-03
Rab protein signal transduction (GO:0032482)	1.97	4.97E-02
mesoderm formation (GO:0001707)	1.96	4.51E-02
mesoderm morphogenesis (GO:0048332)	1.95	4.18E-02
cellular response to calcium ion (GO:0071277)	1.95	1.75E-02
cardiac septum morphogenesis (GO:0060411)	1.94	2.77E-02
cell cycle G1/S phase transition (GO:0044843)	1.93	4.82E-02
interaction with host (GO:0051701)	1.93	2.25E-02
steroid hormone mediated signaling pathway (GO:0043401)	1.90	1.45E-02
regulation of cyclin-dependent protein serine/threonine kinase activity (GO:0000079)	1.87	4.80E-02
synapse assembly (GO:0007416)	1.84	4.99E-02
hair follicle development (GO:0001942)	1.84	4.24E-02

Table S2: **Gene Ontology (GO) enrichment analysis of predicted siRNA off-targets**.
Top 15 biological processes predicted to be affected by the changes in the expression pattern of the targeted genes.

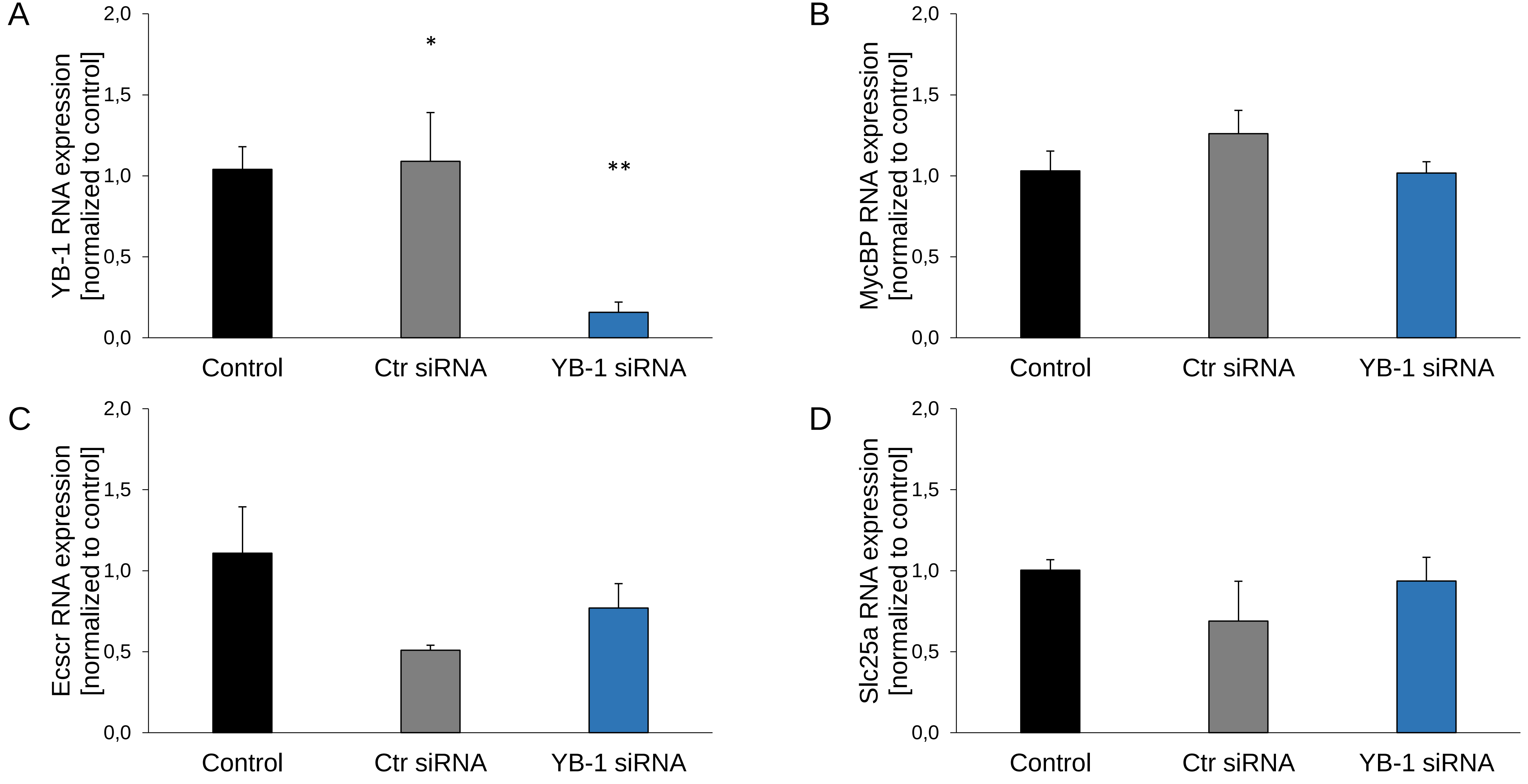

Figure S3: ***YB-1* downregulation in adult rat cardiomyocytes leads to no change in the expression of high-score off-target genes.** Cardiomyocytes were transfected with *YB-1* siRNA or Ctrl siRNA or were non-transfected. RNA expression was quantified after 48 hours (*n* 3 = 5) with real-time RT-PCR using *HPRT1* as the housekeeping gene. (**A**) YB-1, (**B**), MycBP (Myc binding protein) (C) Ecscr (endothelial cell surface expressed chemotaxis and apoptosis regulator) and (**D**) Slc25a (solute carrier family 25 member 23).
